# *PEX6* Mutation in a Child with Infantile Refsum Disease—A Case Report and Literature Review

**DOI:** 10.3390/children10030530

**Published:** 2023-03-09

**Authors:** Ana-Maria Slanina, Adorata-Elena Coman, Dana-Teodora Anton-Păduraru, Elena Popa, Carmen-Liliana Barbacariu, Otilia Novac, Antoneta Dacia Petroaie, Agnes-Iacinta Bacușcă, Mihaela Manole, Adriana Cosmescu

**Affiliations:** 1 Department of Family Medicine, “Grigore T. Popa” University of Medicine and Pharmacy, Iași 700115, Romania; 2 Department of Mother and Child Medicine, “Grigore T. Popa” University of Medicine and Pharmacy, Iași 700115, Romania

**Keywords:** infantile Refsum disease, *PEX6* mutation, peroxisome biogenesis disorder, metabolic anomalies

## Abstract

The aim of this paper is to describe the temporal progression and clinical picture of a 2-year-old child with infantile Refsum disease, as well as the diagnostic procedures performed; this case presented multiple hematologic, metabolic, and developmental complications and progressive disabilities. Genetic testing revealed a mutation of the *PEX6* (Peroxisomal Biogenesis Factor 6) gene, and the metabolic profile was consistent with the diagnosis. Particularly, the child also presented altered coagulation factors and developed a spontaneous brain hemorrhage. The clinical picture includes several neurological, ophthalmological, digestive, cutaneous, and endocrine disorders as a result of the very long chain fatty acid accumulation as well as secondary oxidative anomalies. The study of metabolic disorders occurring because of genetic mutations is a subject of core importance in the pathology of children today. The PEX mutations, difficult to identify antepartum, are linked to an array of cell anomalies with severe consequences on the patient’s status, afflicting multiple organs and systems. This is the reason for which our case history may be relevant, including a vast number of symptoms, as well as modified biological parameters.

## 1. Introduction

Peroxisome biogenesis disorder—Zellweger spectrum disorder (PBD-ZSD) is a rare genetic condition determined by mutations in two copies of any of 13 *PEX* (peroxisome assembly factor-2, PAF-2) genes responsible for the peroxisome function at the cell level. Biochemically, there is an accumulation of phytanic acid in plasma and tissues; it is inherited in an autosomal recessive fashion [1,2]. Infantile Refsum disease is a condition referring to an altered biosynthesis of the peroxisomes [3], featuring multiple biochemical anomalies, including increased plasma levels of phytanic acid, prystanic acid, very long chain fatty acids and biliary acids C27. Patients with Refsum disease are incapable of metabolizing phytanic acid due to the improper activity of phytanoyl-CoA-hydroxylase (PhyH), a peroxisomal enzyme that is a catalyzer of the first step of the alpha-hydroxylation of phytanic acid [1,2].

Fitanic acid replaces other fatty acids, including essential fatty acids such as linoleic and arachidonic acid, at the level of the structures in various tissues [4]. This situation leads to an essential fatty acid deficiency associated with ichthyosis [5]. A gene of Refsum disease, ftanoil-CoA hydroxylase (*PHYH*), has been localized in the band 10p13 between the markers D10S226 and D10S223 [6]. Refsum disease is heterogenous genetically, with up to 55% of cases not being linked to the locus of the *PAHX* gene at D10S547 to D10S223. Some patients present a defect at the perforin 7 level (*PEX7* defect) [7,8,9]. The condition starts during the first year of life and presents with developmental delays, progressive disabilities, visual and hearing problems, and dysmorphic features. Ichthyosis is an unusual symptom [4,5]. The clinical examination typically identifies neurological, ophthalmological, cardiac, and cutaneous signs. Neurologic/ophthalmologic disorders are as follows: partial intermittent sensitive and motor polyneuropathy, cataracts, nystagmus, pigmentary retinitis, anosmia, concentric constriction of the visual field, neuro-sensorial deafness, and modified eye fundus. The signs reflecting cerebellar ataxia are as follows: progressive weakness, gait troubles, and loss of balance. Cardiomyopathy with conduction disorders is a life-threatening sign [4,5]. Liver and kidney dysfunction are clinically silent despite the fatty degenerative evolution. In some situations, there is an ichthyosiform scaling, similar to a mild acquired vulgar ichthyosis, with fine, white scales noticeable on the lower part of the body and also on the limbs. Ichthyosis symptoms may vary from mild hyperkeratosis of the palms and soles to severe scaling of the body. Skeleton disorders (noticed in some patients) are not directly linked to levels of phytanic acid. They appear in 35–75% of cases. 

The aim of this case presentation is to highlight the importance of genetic testing in the diagnosis of rare conditions presenting with multiple apparent non-related pathologies, as well as to underline the effects of altered fatty acid metabolism on different tissues and structures, leading to a generalized disorder involving oxidative mechanisms that need further studies meant to improve the long-term prognosis of the patients.

## 2. Case Report

The child, F.A., a girl aged 2 years, was born from a term pregnancy. The sole prenatal abnormality found was an enlarged 3rd ventricle at a prenatal ultrasound of unknown origin. Her mother was 34 years old, and her father was 35. Both parents were healthy, and the older sister, aged 8 years, was also healthy. There was no previous known history of genetic disorders in the family. The baby was delivered by C-section, with an APGAR score of 8–9 and a birth weight of 3840 g, and was breastfed. At birth, cranial and facial dysmorphism was noticed, with a shortening of the anteroposterior diameter, a round face with a small mouth, enlarged upper eyelids, a retracted jaw, a short neck, and short upper limbs with a unique palmar fold. 

At her general check-up at 2 months, about five violet nodules with a greenish hue were noticed on the lateral side of the thorax, left forearm, left knee, and left shoulder, 5–6 mm in diameter, all of them appearing in the last couple of days. The family physician decided to postpone the 2 months vaccination and recommended coagulation tests (suspecting hematoma) and an ultrasound examination, including an anterior fontanelle ultrasound. However, 48 h later, before concluding the tests, the infant developed seizures and presented emergently in the neurosurgery department. Imaging showed a right temporal cerebral hemorrhage, spontaneously appearing with no trauma as a trigger. Left hemiparesis developed, but the hematoma was under resorption by itself, with surgery thus being avoided. The seizures needed ongoing treatment, and a mild hypertonus of the left part of the body, contrasting with the global axial hypotonia, developed. A motor delay was also present. Secondary epilepsy was diagnosed. As the child grew, there was a major delay in reaching motor and developmental milestones (she did not sit or stand, had no development of speech, etc.). 

### 2.1. Clinical Picture

During the present consultation, the child, aged 2, had a preserved general status, a normal temperature, a weight of 8 kilo (−4 SD), a height of 83 cm, a head circumference of 46.5 cm, and a large anterior fontanelle of 3.5/5 cm (the child had been receiving vitamin D for rickets prophylaxis, the vitamin D serum level being 85 micrograms/dL). Skin and mucosa were slightly pale, presenting an erythematous plaque with scaling on the chin (5 cm diameter) and two hemangiomas on the right side of the thorax and at the left retro-auricular area. Muscle tone was globally reduced, and the subcutaneous tissue was diminished. No palpable lymph nodes were noticed. The examination of the respiratory and cardiovascular systems was normal. The abdominal area was soft on palpation and mobile when breathing, though an enlarged liver was felt some 3 cm beyond the lower rib. The passing of urine and feces was normal. When examining the head, we noticed cranial and facial dysmorphia, with low-positioned ears, a high palate arch, a small mouth, and a mild plagiocephaly.

#### Neurological Examination

At the age of 2 years, the infant was alert, presenting a social smile. She followed what was happening, and her face was symmetric. Mobility was markedly impaired, with a reduced pattern of movements and low muscle tone, though she was able to put her legs together and lift her hands to her mouth or bring them to her median area. The patient did not actively participate when she was lifted from the supine position. She could sit for a few seconds and could hold her head for a while. When lifted from the axillae, she did not support her weight on her legs. When placed on her belly, she lifted and rotated the head. Reflexes are present bilaterally, with no clonus and normal dorsal flexion of the foot. As for cognitive development—a social smile was present; she laughed and inconsistently followed, but there was no speech development. 

### 2.2. Investigations

The first investigation performed for patient F.A. was the FISH test, a postnatal cytogenetic exam, revealing a 46 XX chromosomal formula. At the age of 2 months, the following tests were performed:

- Brain CT with angiography, then contrast-enhanced MRI, with a right temporal brain tissue hematoma of 4.4/2.6 cm being identified.
- EEG while awake and asleep (Neurology Department Obregia Hospital Bucharest)—an asymmetrical baseline pathway with a slower pace and epileptic discharges that were spike-like in the right temporal area was noted.
- Abdominal ultrasound—liver enlargement and an accessory lien were observed.
- Thrombophilia genetic panel—a heterozygote mutation for the MTHFR C677T gene and a homozygote mutation for the PAI-1 4G gene were found.
- Positive antibodies for the cytoplasm of neutrophils (ANCA) were found.
- Antibodies for elastase presented with an increased value.
- Calprotectin presented with an increased value.
- Antithrombin presented with an increased value.
- Protein C and S presented with a low value.
- Coagulation factor X demonstrated a serious reduction of 34% (the existence of Stuart–Prower syndrome was considered). A moderate deficit of the coagulation factors II, V, and VII was identified. The prothrombin time and INR were increased.

Additionally, the following parameters have been tested, with normal values being recorded: homocysteine, circulating immune complexes, B6 and B12 vitamins, apolipoprotein A1 and B, antibodies to anti-leucocytes myeloperoxidase, antibodies for antiprotease 3, antibodies for BPI (bacterial permeability-increasing protein), antibodies for cathepsin G, antibodies for lactoferrin, anti-lysozyme, antibodies to anti-native DNA, ferritin, and D-dimer.

The complete blood count revealed a mild normochromic, normocytic anemia, despite iron supplements being administered. The AST and ALT values were frequently increased, reaching high values in 2022 (1124 and 289 UI/L, respectively).

The ophthalmological check-up revealed an opacified crystalline lens, a compost hypermetropic astigmatism, and divergent strabismus. The audiology consultation showed a medium bilateral neuro-sensorial hypoacusis. A heart ultrasound was performed, and an atrial septal defect of small dimensions was present, as well as a mild tricuspid reflux.

An endocrinology check-up in November 2021, including evaluation of the ACTH and cortisol levels and a stimulation test at Synacthen, confirmed the existence of a mild suprarenal deficit.

At the age of 9 months, genetic testing was performed by WES Centogene in Germany. The *PEX6* variant c.1445_1448del p. (Ala483del) was identified; this is an in-frame deletion of 3 bps in exon 6, which causes the loss of residue Ala at position 483. ClinVar lists this variant as uncertain (clinical testing. Variation ID: 558266). The second modified *PEX6* variant c.166G>C p. (Ala56Pro) causes an amino acid change from Ala to Pro at position 56. It is classified as a variant of uncertain significance (class 3). Double-stranded DNA capture baits against approximately 36.5 Mb of the human coding exome (targeting >98% of the coding RefSeq from the human genome build GRCh37/hg19) were used to enrich target regions from fragmented genomic DNA with the Twist Human Core Exome Plus kit. The generated library was sequenced on an Illumine platform to obtain at least 20× coverage depth for >98% of the targeted bases. An in-house bioinformatics pipeline, including read alignment to the GRCh37/hg19 genome assembly, variant calling (single-nucleotide and small deletion/insertion variants), annotation, and comprehensive variant filtering, was applied. The investigation for relevant variants was focused on coding exons and flanking plus/minus 20 intronic nucleotides of genes with clear gene-phenotype evidence (based on OMIM information). All potential modes of inheritance patterns were considered.

Both parents were heterozygotes for the gene *PEX6*. Biochemical metabolic determinations needed for the peroxisome disease (VLCFA, prystanic acid, phytanic acid (3 mg/L), and serum plasmalogen showed high values for VLCFA C26 and pipecolic acid (406.9 µmol/L). There was also a modified ratio of VLCFA C24/C22 and C26/C22. As a consequence, the diagnosis of the *PEX6*, 4B peroxisome disease, infantile Refsum disease (PBD-ZSD), was established.

### 2.3. Diagnosis and Associated Conditions

In addition to the diagnosis of infantile Refsum disease (PBD-ZSD) due to a *PEX6* mutation of uncertain significance, our patient presented with several secondary conditions, mostly related to the biochemical alteration of fatty acid metabolism. The cerebral hemorrhage led to secondary epilepsy, while the gross motor and developmental delays were caused by metabolic encephalopathy with cerebral ataxia, with a demyelinating process being suspected. An ophthalmological examination confirmed the presence of bilateral cataracts, divergent strabismus, and hypermetropic astigmatism. A medium bilateral neuro-sensorial hypoacusis was also present. From the cardiology perspective, the heart seemed unafflicted, except for a minor atrial septal defect. A major issue stands in the digestive area, where metabolic liver disease is present, with hepatomegaly and high liver enzymes, all other possible causes being excluded. Inflammatory bowel disease with malabsorption is also possible, considering the elevated level of calprotectin. Due to the ANCA titer being modified, an autoimmune mechanism may be suspected as an additional factor involved in this area, as well as in the skin and blood disorders. The child presented with scaling eczema of the chin, as well as skin xerosis consistent with Refsum syndrome, in addition to osteo articular signs, such as the delayed closure of the anterior fontanelle. Of particular interest, with no previous recording in the literature, is the coagulation disorder because of the homozygote mutation for the PAI-1-4G gene and the heterozygote mutation for the MTHFR C677T gene, as well as the Stuart–Prower Syndrome with a reduced level of Factor X. A particular situation refers to the mild suprarenal deficit identified by the endocrinology team.

## 3. Discussion

**Figure 1:**
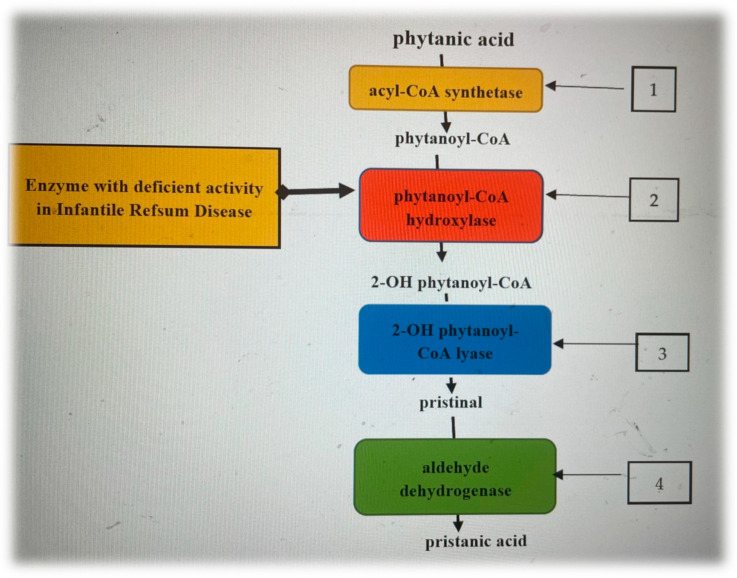
Phytanic acid enzymatic steps of alpha-oxidation [12,13,14]. 1. On the cytosolic side of the peroxisomes, phytanic acid is activated by an acyl-CoA synthetase, resulting in phytanoyl-CoA, which is transported into the peroxisome by a specific transporter protein. 2. Phytanoyl-CoA is oxidized by phytanoyl-CoA hydroxylase, producing 2-hydroxyphytanoyl-CoA. 3. 2-hydroxyphytanoyl-CoA is then dehydrogenated by the enzyme 2-hydroxyphytanoyl-CoA lyase, resulting in pristanaldehide. 4. Pristanaldehide is oxidized by pristanal dehydrogenase, which converts it to pristanic acid.

Compared to most normal fatty acids, which are degraded by β-oxidation, the catabolism of phytanic acid starts with α-oxidation with the formation of pristanic acid (Figure 1), which is subsequently metabolized by the β-oxidation pathway [10,11]. Patients with Refsum disease have a mutation in the key enzyme of α-oxidation, and consequently, an abnormal accumulation of PA occurs [10].

Phytanic acid and pristanic acid have been shown to potently activate multiple subtypes of peroxisome proliferator-activated receptors (PPARs) [10,15]. PPAR are ligand-activated transcription factors involved in the regulation of carbohydrate and lipid metabolism, energy homeostasis, inflammation, and immunity [16,17,18]. PPARs are molecular therapeutic targets for metabolic diseases, such as diabetes, dyslipidemia, atherosclerosis, and chronic inflammation [17,18]. These findings suggest that PA and PrA have a double role: a risk factor for patients with Refsum disease and possible beneficial effects in subjects without genetic alterations of phytol-derived fatty acid metabolism.

Patients diagnosed with infantile Refsum disease have defective phytanic acid metabolism and may have severe clinical complications such as growth retardation and neurological and cardiovascular abnormalities. Dhaunsi et al. [19] showed that phytanic acid impaired IGF-1-induced DNA synthesis in smooth muscle cells, suggesting its role as a growth retardant in the vascular system (Dhaunsi et al.) [19]. The role of nitric oxide NO at the vascular level is also known. Thus, the excess production of nitric oxide NO inhibits vascular growth in vivo and in cell cultures (Dhaunsi, G, Zuckerbraun BS et al.) [19,20]. Moreover, concentrations exceeding normal values of phytanic acid enhance nitric oxide production in vascular smooth muscle cells and induce apoptosis (Dhaunsi et al.; Idel et al.) [20,21].

The presented complications are due to the coagulation disorder with hemorrhagic risk. The metabolic overview shows a decline in growth and the presence of episodes of anemia; the liver condition must be carefully monitored due to the oscillating high liver enzymes levels. Last but not least, motor and mental retardation, hearing and sight problems have a major impact on long-term development. 

Case management of infantile Refsum disease (IRD) is multidisciplinary, but treatment is still only symptomatic (Sa) [22]. Reports on the impact of treatment on disease progression are limited, probably due to the small number of cases. However, some research [Sa, Moser] has shown that dietary changes lead to specific biochemical effects. Further studies are needed to evaluate the impact of a low-phytanic-acid diet in the recommended management of patients with IRD [22,23].

To improve the motor development delay, the pediatric neurologist recommended types of kinesiotherapy used in treating central nervous system locomotor disorders. For the control of seizures, the child received treatment with levetiracetam, carbamazepine, and clobazam, all dose-adjusted for her weight. The child is undergoing an assessment conducted by a team including a nutritionist, an endocrinologist, a genetic specialist, a gastroenterologist, and a neurologist. The aim is to create a complex rehabilitation plan to correct the weight deficit and the liver condition and to control the excessive synthesis of phytanic acid (avoiding green vegetables and animal fat). The plan includes genetic consultations, yearly ophthalmologic and audiology consultations, neurology consultation every 6 months, yearly gastroenterology consultation, C27 bile acid measurement, supplementation with vitamins A, D, E, and K and DHA, yearly endocrinologic and nephrological consultation to evaluate the risk of oxalate calculi formation, regular dental consultation, and DEXA measurement for bone frailty.

### Antenatal Diagnosis and Gene Therapy

Genetic counseling (testing and family planning) is recommended because infantile Refsum disease is inherited in an autosomal recessive manner [Kumar, Waterham HR] [24,25]. Prenatal screening can be performed in the first or second trimester using biochemical or genetic testing of cultured amniocytes and chorionic villus sampling for very-long-chain fatty acids and plasmalogen synthesis (Braverman, 2016; Al-Sayed) [26,27]. Preimplantation genetic testing is one of the disease prevention options for couples affected by infantile Refsum disease (Braverman, 2016; Al-Sayed) [26,27].

In the long term, gene therapy may be the treatment of choice, but many issues must be resolved before it can be applied (safety and effectiveness). Currently, the potential of enzyme replacement therapy (ERT) similar to that for lysosomal storage diseases is under investigation (Waterman HR) [25]. Gene therapy is an exciting area for many genetic disorders and may offer hope for patients with PBD [24,25,26,27,28].

## 4. Conclusions

The *PEX6* mutations, in our case, are responsible for a large range of abnormalities, as well as for metabolic complications involving different structures and tissues. Pristanic and phytanic acids exert different toxic activities on brain cells via the disturbance of Ca2+ homoeostasis and interference with mitochondrial functions. Pristanic acid exerts a stronger toxic activity on brain cells than phytanic acid [29]. Altering structures in the neuronal pathways as well as of the liver tissue is a subject of further study, especially concerning the effect of the phytanic acid on the mitochondrial function and on increasing the reactive oxygen species. Altered fatty acid metabolism is involved in the dysregulation of inflammation and immunity via multiple subtypes of peroxisome proliferator-activated receptors (PPARs). The role of NO and IGF-1 in apoptosis and vascular proliferation may be the subject of detailed research. The activation of the autoimmune mechanism in the genesis of pathological mechanisms underlying the clinical picture of Refsum syndrome is also of interest. Particular for this case, is the coagulation disorder leading to a spontaneous brain hemorrhage. The case prognosis is reserved considering the conditions afflicting the patient and the scarcity of literature data (60 cases all over the world) (Schwartz RA) [30], as well as the limited therapeutic options for this rare genetic condition.

## Data Availability

Some of the unpublished data may be protected due to privacy or ethical restriction.
